# The Barriers of the Assistive Robotics Market—What Inhibits Health Innovation?

**DOI:** 10.3390/s21093111

**Published:** 2021-04-29

**Authors:** Gabriel Aguiar Noury, Andreas Walmsley, Ray B. Jones, Swen E. Gaudl

**Affiliations:** 1 School of Engineering, Computing and Mathematics, University of Plymouth, Plymouth PL48AA, UK; swen.gaudl@plymouth.ac.uk; 2 International Centre for Transformational Entrepreneurship, Coventry University, Coventry CV15FB, UK; ad3412@coventry.ac.uk; 3 School of Nursing and Midwifery, University of Plymouth, Plymouth PL48AA, UK; ray.jones@plymouth.ac.uk

**Keywords:** barriers to innovation, assistive robotics, market barriers, healthcare innovation

## Abstract

Demographic changes are putting the healthcare industry under pressure. However, while other industries have been able to automate their operation through robotic and autonomous systems, the healthcare sector is still reluctant to change. What makes robotic innovation in healthcare so difficult? Despite offering more efficient, and consumer-friendly care, the assistive robotics market has lacked penetration. To answer this question, we have broken down the development process, taking a market transformation perspective. By interviewing assistive robotics companies at different business stages from France and the UK, this paper identifies new insight into the main barriers of the assistive robotics market that are inhibiting the sector. Their impact is analysed during the different stages of the development, exploring how these barriers affect the planning, conceptualisation and adoption of these solutions. This research presents a foundation for understanding innovation barriers that high-tech ventures face in the healthcare industry, and the need for public policy measures to support these technology-based firms.

## 1. Introduction

Technology has always been a vital ally for healthcare [1], from the invention of new diagnostic capabilities and therapies to practices that improve the overall quality and cost-effectiveness of the care delivery system. This has changed healthcare and the perception of healthcare. More generally, technology is considered the ‘holy grail’ of public policy, achieving outcomes at a lower cost [2]. Nevertheless, the healthcare sector and its suppliers are not as productive as they could be at adopting new technologies.

This disparity is generally attributed to the market barriers present in the sector that are impeding the adoption of innovations and therefore their spread. Innovations in healthcare do not follow a linear pathway even though there is still an inclination to see these as such [3]. Indeed, the innovation process in healthcare has been described as long, incremental and path-dependent [4], reflecting [5] recognition of the co-evolutionary and path-dependent nature of innovation processes more generally.

Innovations today are desperately needed. Healthcare systems worldwide are under increasing pressure. Life expectancy has increased, from 52.6 in 1960 to 72.0 in 2016 [6], and the last two decades have witnessed continued growth in the global population [7]. In the European Union, despite fertility levels being below what is generally regarded as the level necessary for a population to maintain its size [8], it is estimated that population levels will remain relatively static (falling approximately 0.35% by 2050) due to net migration [9], while life expectancy has increased by 2.9 years, from 77.7 in 2002 to 80.6 in 2015 [10].

For the healthcare sector, the ageing of the population translates into many more individuals with one or more long-term conditions, and more complex patterns of diseases [11]. While shortages of qualified carers are continuously increasing [12], because of increased constraints on the funding and resources available, governments are urged to find alternatives to help close these gaps. To provide a more tangible example, more than 9 million people in the EU need help getting out of bed [13], and this figure is likely to increase. A new wave of innovations is needed if we want to provide people with affordable and effective healthcare [14].

Based on these previously introduced challenges, the assistive robotics (AR) market is surging, providing support to patients and care workers, in the globalisation of healthcare. Assistive robots are shifting the model of healthcare from one focused on hospital treatment to one that supports independent life [13]. However, despite the developments of the last two decades, the AR market is not having the impact expected due to the current market barriers [15].

Identifying obstacles to innovation has clear policy relevance, since addressing these barriers could increase the population of innovators and boost the performance of current ones [16,17,18]. Previous research has called for more studies on problems and challenges surrounding innovation processes in healthcare settings [19].

By offering insights into the current barriers of the AR market, we seek to assist this process for a market we have not seen before. The development of assistive robots for the healthcare sector is not only an issue of technology or societal acceptance. We need to work on the broader market barriers and ways to overcome them. Only then, entrepreneurs and firms will be aware of the venture challenges, and governments could take measures to support these inventions that are highly needed [20].

Consequently, this paper starts by giving an overview of the current state of this market in Section 2. Section 3 presents the methodology. Then, drawing on the experience of 17 firms engaged in the AR market, Section 4 explores the primary market barriers relating to the product development process, from conceptualisation to commercialisation. Next, we discuss the barriers and its combined effect while providing implications for policy measures to support technology-based firms. Finally, concluding remarks and implications for research are provided in Section 5.

## 2. Background

The AR market relates to robotics and autonomous systems with the primary role of providing assistive help to carers or directly to patients in hospital, specialist care facility or domestic healthcare settings [13]. This market excludes clinical robots (e.g., surgical robots, [21,22]; robots for diagnosis [23] or training purposes [24]) and rehabilitation robots (e.g., prosthesis and exoskeletons [25] and rehabilitation systems [26]). It also excludes the sector known as service robots or robots for domestic tasks (e.g., vacuum or window cleaning, lawn mowing) [27].

**Figure 1:**
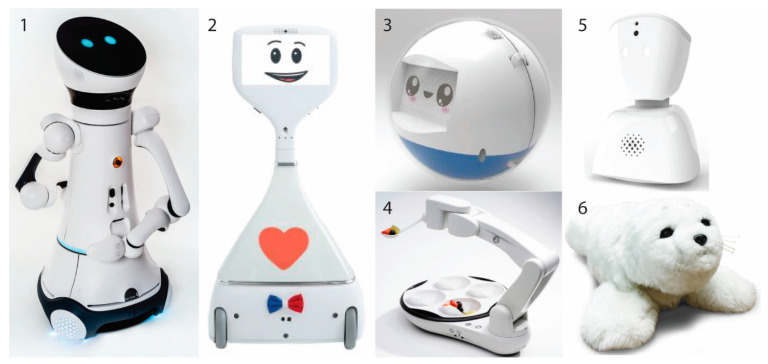
Assistive robots available in the market. (**1**) Care-O-bot; domestic robot assistant, (**2**) Cutii; telepresence robot for older people, (**3**) Leka; a therapeutic robot for children with developmental disorders, (**4**) Obi; robotic feeding device, (**5**) AV1; robotic avatar for users with long-term conditions, (**6**) Paro; therapeutic robot.

The AR market features robots that organise and deliver medication [28], that support activities of daily living such as eating [29] or putting clothes on [30], and that can improve hygiene [31] or the recovery process [28]. It also includes lifting and displacing aids, from helping nurses moving the patient from bed to wheelchair or helping the elderly move around their homes [32]. Socially assistive robots are also part of this sector, offering patient aid in therapy rehabilitation [33]. For instance, the best-known example, Paro, is a therapeutic seal for people with dementia [34]. Finally, AR also includes robots for the transportation of drugs, food or other resources [35], and communication purposes [36] (Figure 1). It is recognized that this list covers just a fraction of the various applications and opportunities for which assistive robots are being developed in universities, research labs, and start-up companies [37]. Figure 1 shows some visual example of AR robots.

The assistive robotic market is different from any software or hardware application, or even other AR technologies. Manufacturing or agricultural robots have required a focus on the design of the mechatronic structure. Aerial, marine and transportation robots have focused on autonomous navigation and object detection. On the other hand, assistive robots build around the nascent human–robot interaction field [38]. They are robots that interact physically with their user, continuously and autonomously monitoring their condition, while supporting them in different activities. The design involves different stakeholders, different structural systems abilities and technologies, different design processes, and different testing methodologies, and as a result, it produces different market barriers for the suppliers of this emerging industry [37,38,39].

The size of the global AR market in 2015 was over USD 200 million, with an 18.9% compound annual growth rate estimation from 2016 to 2024 [40]. It is characterised as a highly fragmented sector, driven by some small players [41]. The industry is in a nascent state, where start-ups are pushing with an increasing number of robotic products that perform a wide variety of tasks [41].

However, despite the potential for the technology, there is limited evidence of AR projects that transform into new products and services for the user [38]. The International Federation of Robotics registered only around 5305 robotics solutions for supporting elderly and handicapped sold in 2016 in the world [27].

Furthermore, from February 2019 to March 2019, we contacted 11 robotic companies in the field, inquiring their trending activities (Table 1). Only one was currently supplying to the European market, and three were trading outside the EU. One of these 11 businesses, the American company Jibo Inc., who raised nearly USD 72.7 M with his social assistive robot [42], was forced to sell its assets in December 2018 and has been out of business since then.

### Innovation through Barrier Identification

Innovation research may be divided into two general areas of analysis: an economic-oriented tradition and an organization-oriented tradition [43]. The first studies innovation across countries and industrial sectors; for instance, through the sourcing of knowledge for innovation [44], while the latter focuses on how specific new products are developed; the structure and process by which organisations create products. This study focuses on the latter since by analysing the product development process and identifying the innovation inhibitors or barriers; it is possible to take action to eliminate them and restore the ‘natural flow of innovation’ [45]. This approach is particularly useful for cutting-edge technologies and new markets [46], such as the AR sector.

Market barriers to new technologies can be perceived in different ways [45]. For instance, through the research, development and deployment perspective, we emphasise the nature of the technology, its manufacture and user adoption [47,48]. Since this represents the first line of the development process, much of the literature available around case studies of AR focuses only on these elements of innovations. For instance, [49,50] or [51] studied user needs, acceptability and usability of innovations in technology with end-users. Numerous studies outline the critical technology targets for AR, drawing roadmaps for the development of this sector and main research challenges (e.g., [13,28,38]).

None of these studies consider the development of assistive robots as a market-orientated process; the obstacles of producing the technology from concept design to the commercialisation of the AR. We argue, therefore, that since the application of robotics in healthcare is not only an issue of technology or societal acceptance, but special attention has also to be paid to broader market barriers and ways to overcome them. As a result, this paper aimed to address the following question: what are the market barriers that are inhibiting the development of AR?

To thoroughly understand the barriers, we have explored them from a market transformation perspective. Here, we do not focus on the nature of the technology or typical operating characteristics of conventional markets, but rather on the development of technology as part of a market process and what needs to be carried out in practical terms to create markets for new technologies [47]. To date, most of the relevant literature centres on market transformation studied in the eHealth sector, i.e., the use of information and communication technologies in healthcare (i.e., [52,53,54]).

There is, therefore, a predominant focus only on software products. A distinction exists between the development of a hardware product and its commercialisation. Consequently, market barriers may also be different [55,56].

This study seeks to apply the Painuly (2001) framework, which builds upon the barriers approach to innovation, focusing on a market transformation perspective [57]. Painuly developed a framework for analysing the barriers to renewable energy penetration when this was an emerging sector. The study analysed the obstacles to creating a new and innovative market. The framework provides a methodology for identifying barriers by reviewing case studies, including criteria for selection and measures to overcome the barriers identified.

**Figure 2:**
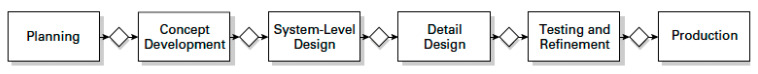
Eppinger, S. D. and Ulrich’s product development process. Source; [58].

Different models describe product development processes for different industries. Ulrich and Eppinger’s (2016) six phases of product development balance hardware and software development in the product development process (Figure 2) [58]. The process includes the tasks and responsibilities of the critical functions: marketing, design, and manufacture. This model, along with Painuly’s (2001) framework, will serve as a basis of our exploration of inefficiencies in the AR market.

To summarise, using a market transformation perspective, this paper explores and identifies market barriers as perceived by companies engaged or trying to engage in the AR market. We argue the study is particularly pertinent at a time of growing care needs, coupled with a minimal understanding of the AR market and the implications for health innovation policy. The current literature focuses on soft elements of AR innovation and not on the implications of the market deployment of these technologies.

Moreover, the current literature around market barriers for healthcare focuses only on digital technologies. The AR market is fundamentally different from the digital or any other robotics sector. Therefore, only by exploring market barriers will policymakers and entrepreneurs be aware of the venture challenges and take measures to support these inventions that are highly needed for the healthcare sector.

## 3. Methodology

Semi-structured interviews were conducted with 17 people from different assistive robotic companies. The companies were based either in the UK or France (Table 2). The interviews involved the owners of the company, wherever possible. Where this was not possible, interviews were conducted with senior managers.

The study explored their perspectives relating to the challenges they perceived and were facing during the product development and commercialisation process. Apart from having to be involved in AR, the other criterion used to select businesses was the age of the business. We aimed to obtain companies covering different business stages: seed and development, start-up, expansion, and maturity.

We interviewed start-ups and companies working from at least six months to up to 11 years. This was intended in order to map the different obstacles assistive robotic companies face during the market process/product development and life cycle. Sampling comprised a mixture of purposive and convenience, given that companies had to meet our selection criteria [59]. Participants were briefed about the nature of the study, participation was entirely voluntary, and it was agreed that companies would not be mentioned by name.

To design and structure the questions for the interview, we drew on Ulrich and Eppinger’s (2015) six phases of product development. The process includes the tasks and responsibilities of the critical functions of the company for each phase, including marketing, design, and manufacture. The linearity of the process allowed us to structure the interview, but it did not unduly restrain or influence the conversation. In this sense, interviews were semi-structured and akin to Kvale’s notion of conversations with a purpose [60]. For each of these stages, several questions were elaborated.

The questions were designed following the study objectives: explore AR market barriers from the perspective of the companies, the critical functions of the development process and an initial literature review from AR projects [61]. Examples of questions include ‘What was the most difficult part during the phase of product planning?’, ‘What was the biggest challenge in assessing customer needs?’, ‘Tell me about the problems you faced during your prototype testing phase’. Follow up questions were asked to explore the participants’ answers in more depth.

Interviews lasted between 20 and 50 min and were audio recorded. They started with an introduction of the objectives of the study and by the participant’s overview of their business.

Then, interviews were transcribed using IBM Watson Speech to text. The transcripts were cleaned and put into a standardised format. The general inductive approach was used to analyse the transcripts to identify themes in the text that were related to the study evaluation objectives [61]. The analysis started with a close reading of the text, and the themes were developed, which in the view of the investigators captures core messages reported by participants, particularly around barriers and market inefficiencies. Overlapping and redundant themes were reduced through a search of subtopics, including different points of view and new insight. To show when companies perceived the effects of the barriers, we also classified themes according to their appearance in the product development process. Nineteen significant themes were identified, hereafter referred to as barriers, and are described in the next section.

## 4. Discussion

**Figure 3:**
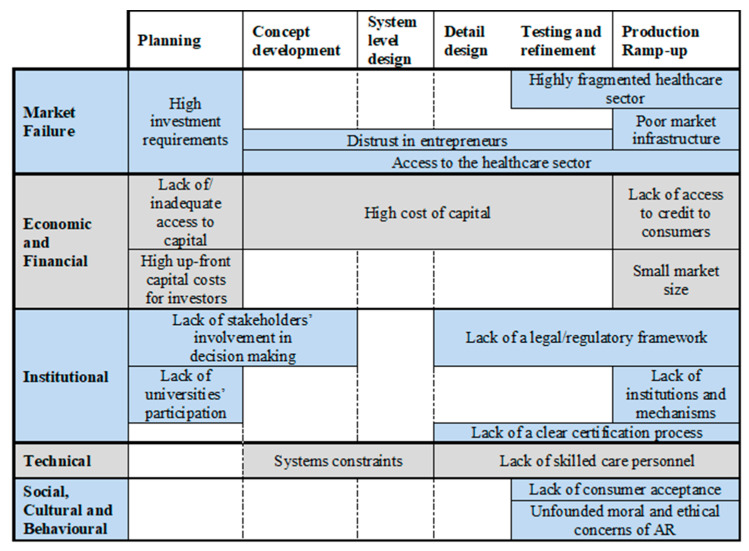
Barriers over the development process of AR for healthcare.

The present paper follows [57] for classifying the nineteen themes identified. Following the Painuly categorisation system, we organise the themes into five distinct sets of market barriers, shown in Table 3. Besides, by his classification scheme, Table 4 shows the elements or the leading examples of the barrier impact in the AR market. Figure 3 displays barrier emergence during the product development process.

These tables present a useful heuristic device as a means of understanding the nature and scope of critical barriers in the AR market, as evidenced in the interviews. However, we recognise that the classification of barriers shown in Table 3 is not rigid, that some of the barriers are interrelated, and that some barriers can, arguably, belong in more than one category and share a similar impact. That is why the remaining section discusses and analyses these findings not as individual and independent elements, but as members of each barrier category.

### 4.1. Market Failure

This refers to the lack of conditions needed for perfect competition in the market, most notably access to information. The impact of hereof can be seen through the whole development process.

#### 4.1.1. Access to Highly Fragmented Healthcare Sectors

The most frequent barrier identified was access to the healthcare industry. Specifically, for innovation to take place, entrepreneurs need access to patients, families and carers’ needs, to know and understand their problems. Only with this knowledge is it possible to generate and implement ideas for new improvements. User-centred design is seen as a vital tool when it comes to AR innovation for healthcare, and its input could overcome further barriers of adaptability and implementation. The interdisciplinary field of the innovation process demands specialised knowledge in methods of care, presenting new challenges for innovators who ask themselves whom they should involve in the process. Being able to define and then access an ideally representative sample of early adopters for the market is the initial challenge that AR companies face.

*‘Without contacts, there is not a really a way into it […] you are not exactly going to be able to walk into any care home ask them; do you want a robot? Can we now work with you?’*

The fragmented structure of healthcare systems was seen as the primary reason for this barrier since there is not a clear go-to point. Take, for instance, the National Health Service (NHS) in the UK. Commonly seen as one entity, it is, in fact, a group of many individual organisations: NHS England, NHS Scotland, NHS Wales, and the affiliated Health and Social Care in Northern Ireland, Public Health England, 195 Clinical Commissioning Groups (CCGs), 245 hospital or acute trusts, around 7454 general practices only in England, and thousands of community providers. Each often includes clinicians, financial managers, commissioners and Information Technology managers.

Respondents mentioned that some health organisations charge for preliminary assistance, even up to *‘GBP 1500 for a 3 h consultation’*. Besides being an elevated price for new ventures, 3 h is unrealistic about the actual time needed to gain sufficient understanding of consumer needs. Every decision taken during the innovation process should count with the feedback of lead-users and stakeholders involved. Some companies mentioned that for conceptualisation, development and testing of their prototype product, consultation time ranged between 10 and 15 h.

The alternative is to look to family and friends to become involved in the process. This is a reason why so many entrepreneurs innovate around the needs of their relatives. Participants also mentioned the extreme option of having to ‘*look in the streets*’ for potential lead-users.

Distrust towards entrepreneurs was also mentioned as a barrier. Participants mentioned there was an expectation for eHealth entrepreneurs to have an extended portfolio of developing robots as if they were app developers. Therefore, many mentioned that *‘doors are not open to entrepreneurs with good ideas’*.

*‘We don’t have a reputation, or much more, products to our names, so that we can go and say, this is a current problem, look what we have done, we got the solution … no one listens’.*

In particular, this last quotation illustrates the credibility problem these new entrepreneurs have in gaining acceptance in the eHealth marketplace.

What makes the mentioned lack of access different for AR companies compared to digital companies is its combination with social, cultural and behavioural barriers. Where digital technologies also suffer from negative attitudes and beliefs from the healthcare members, individuals have been widely exposed to apps and programs in their daily lives, greatly improving the user perception of the innovation and adoption of these solutions [62,63]. Robots, on the other hand, have not yet had the same visibility. Combined with this distrust towards entrepreneurs, we have a market that closes its doors to technology and innovation—driven in part by ignorance, including uninformed ethical concerns as well as cultural barriers. This restriction not only impacts the development process but also businesses’ access to funding opportunities since cost-saving studies cannot be carried out without healthcare support.

#### 4.1.2. Complex Market Infrastructure

The complex structure of the healthcare system has a direct impact on our target market infrastructure. According to interviewers, multiple people influence the procurement process, making it challenging to identify and locate who is, ultimately, responsible for making the final decision on a purchase or commission. Interviewers also mentioned that the spread of AR technologies within healthcare systems is slow and fails to achieve widespread use.

The number of individuals involved in the adoption process not only defines the demand of the market but also slows down the diffusion of a product. It also raises a fundamental problem: transparency regarding the structures and purchasing processes within each organisation. This barrier has further impacts that discourage private funding from supporting AR ventures.

*‘[regarding the purchasing processes] for someone that is new to the market it is exceptionally difficult to get to the right people, to go to the people that make the choices’.*

The lack of a technological appraisal tool or method for AR that could benefit companies as a route to widespread use was also mentioned. There is currently a market search friction problem; purchasing and supply agencies do not know what technologies are currently available. There is no standard platform for AR to prove their benefits to healthcare stakeholders and look for potential buyers. Moreover, there are no distribution channels for AR technologies.

*‘it is quite hard to reach the client, and distributors ask for a lot of money, raising prices’.*

Currently, initiatives such as Innovation, Health and Wealth, introducing a legal obligation on all UK’s CCGs to offer National Institute for Health and Care Excellence (NICE) approved technologies to patients, have a positive impact on new medicine use, but it does not cover non-medicines [2]. Therefore, initiatives such as those should be designed to overcome search friction.

### 4.2. Economic and Financial

This category describes those barriers which had an impact on the access to finance and the conditions attached to obtaining financial backing. From the interviews, it was clear that the main barriers focused on the lack of seed funding with implications for scalability of the market. These issues have a significant impact during the first stages of the development process (Figure 3).

#### 4.2.1. Capital and Investment

According to interviewees, on average, three to four prototypes are required before obtaining a minimum viable product. This could translate into four years of work and at least three rounds of funding. This demonstrates that it is not only the technical challenges that discourage AR innovation but also economic viability considerations. The fear of financial failure appears to be thwarting most projects before they properly have the chance to flourish.

As in other markets, robotics companies frequently start with entrepreneurs working part-time on their projects. However, while app developers can find solutions to avoid incurring initial costs, for robotics start-ups, the scope here is more limited. A high initial investment is needed for buying off-the-shelf devices, as well as fast prototyping tools.

*‘Finance, is so difficult, is not cheap, is an expensive journey, and this stops people from doing it’.*

Several participants explained that there is an underdeveloped capital market, scarcity of capital, restricted entry, unavowed regulations and lack of access to affordable capital for AR ventures. Others mentioned that there were few investors and venture capital providers who understood hardware development and therefore, the seed capital needed. Those who are willing to invest perceive this as a high-risk involvement, demanding high-interest rates to offset the risks they take.

There is poor creditworthiness for AR and inadequate recovery regulations. Without a repayment history, credit score or available assets, financial institutes perceive early-stage AR companies as a high-risk investment. This translates into high interest rates and low credit limits. This has a direct impact in particular on indebted businesses. AR companies are often forced to recover through the sale of the collaterals, which in this market is the manufacturing equipment or the technology inside the robot, not allowing a real recovery for start-ups.

The lack of openness from the healthcare sector makes it difficult for start-ups to prove cost-savings of their products, how they affect the right treatment and the downstream healthcare system. Besides, assistive robots have an impact on the quality of life of patients (i.e., therapeutic robots for reducing isolation, stress, anxiety), making it difficult to define the real value of innovation without standard technological appraisal tools. Additionally, participants’ views were that health commissioning bodies take their investment decisions motivated more on cost and risk concerns than the healthcare outcomes for patients.

However, it was also noticed that companies interviewed were not aware of funding opportunities provided by the European Union through their various initiatives (e.g., [64]). Further research is needed to understand whether or not companies perceived these EU initiatives as convenient for their business development, or whether there are blockers or challenges for companies to access these funding opportunities.

#### 4.2.2. Customer Credit Facilities and Market Size

The cost of acquisition and use of assistive robots was reported as a significant barrier. Business to consumer schemes are not viable for most companies since final AR products are commonly too expensive for their end-users due to the costs associated with manufacturing and the technology behind the product. Even if consumers want to purchase AR products, because of under-developed credit facilities, this could present a barrier. Thus, currently, there are no government policies, strategies and incentives for encouraging the adoption of AR technologies that extend to offering either credit facilities or other elements of financial support.

On the other hand, due to the complex structure of healthcare systems, the end-user generally has no input on pricing considerations, only purchasing and supply agencies. Therefore, if the product has not been prescribed, the final user will have to pay the full price for a product, irrespective of affordability. All of this has an impact on the current market size for AR companies.

Healthcare systems around the world not only vary in their structures and entry channels but also in terms of their regulations and economic context, different leadership styles and environments. Product or service development strategies may need to be tailored to each unique system. This makes access to new markets, i.e., ones with which the eHealth entrepreneur is not familiar, difficult. Interview responses indicated that companies from France and the UK did not have any knowledge about the other’s healthcare market, and both mentioned that they could not enter a new market without support. Some companies mentioned difficulties expanding to other regions within the same country due to the fragmented structure of the healthcare sector.

### 4.3. Institutional

Since AR in the healthcare sector represents an emerging market, policies and governments are still playing a catch-up game concerning regulation and support. We refer here to the notion of institutional burdens, that is underdeveloped or absent institutional structures which can hinder aspiring entrepreneurs from exploiting opportunities fully [65].

#### 4.3.1. Poor Legislation, Poor Policies

The AR market lacks specialised agencies at a planning level that can develop and ensure a safety adoption framework for AR innovations. As mentioned in Section 4.1.2, there is lack of transparency in the purchasing and adoption procedure of new technologies for the healthcare sector, starting with initial go-to points for entrepreneurs. The absence of adequate appraisal tools makes it difficult for start-ups to assess the economic evaluation of their products, which generally have an impact on the quality of life of patients (i.e., therapeutic robots for reducing isolation, stress, anxiety).

In the same way, there is no education for health stakeholders of the technology available and opportunities this new market presents. Besides, liability concerns interrupt the development process and AR adoption. All of this is compounded by a missing regulatory body that supports early-stage companies, regulates the development process, and promotes the adoption of assistive robots.

A lack of planning and the absence of policies to foster the development of innovations in the eHealth sector was also mentioned. Barriers such as access to health experts and lead users could be overcome with the right regulatory body. It could also address the absence of legislation surrounding liability while testing or adopting new AR products at the organisational and health professional levels. This includes critical appraisal tools for testing AR, approved by the relevant bodies.

These barriers are the result of the reduced involvement that the supply side has in decision making. Different from traditional markets, the healthcare stakeholders’ counterparts do not involve engineers, developers, and entrepreneurs in the development of policies for innovations. There is currently a consultation culture missing, driven by a tension surrounding change alongside social, cultural and behavioural barriers, described in Section 4.5.

Another essential subject mentioned was the lack of support for entrepreneurs to protect the intellectual property (IP) of their products. The current process takes around five years to complete and requires a substantial investment in applications for patents and design rights. In the UK, only one in twenty applications get a patent without professional support. Subsequently, this might need to be renewed on an annual basis. This problem prevents companies from accessing quality manufacturers recognised by the healthcare industry, which often requires IP before getting involved in the process.

#### 4.3.2. Lack of University Participation

Most companies that took part in this study mentioned the vital role that universities played in this market, from access to technical and medical knowledge to the recruitment of new talent. Nevertheless, it was acknowledged that doors are not open to entrepreneurs and businesses.

*‘You can’t put a price [university support] but, unfortunately, they are not interested in product development’.*

Moreover, current policies from some of the universities mentioned during the interviews highlighted the regulations against university spinout. Companies believe that some universities currently retain intellectual property over any technological development. Therefore, if an entrepreneur wants to apply the research carried out, they will have to address the corresponding payback to the university.

Universities’ investment and support for AR companies is visible on launchpads and incubators. Besides, previous studies have evidenced that IP-based spin-offs are an ideal mechanism for technology transfer [66]. However, following the interviewers’ answers, universities should be more flexible about their current IP policies towards spin-offs. Therefore, further research is needed to provide fair public policies for all the parties involved [67].

#### 4.3.3. Lack of a Transparent Certification Process

Navigating the tricky channels to obtain medical certification was regarded by the businesses interviewed as burdensome and costly.

A device must receive a Conformitè Europëenne (CE) mark as a medical device before being used in the healthcare sector of the European Economic Area [68]. In the U.S., the Food and Drug Administration (FDA) regulates the sale of medical device products [69]. Certification is mandatory, and it is the manufacturer’s sole responsibility to obtain approval. Overall, to obtain a certification, the manufacturer must follow conformity assessment procedures depending upon the classification of the medical device. Several factors are considered, such as usage time, lifespan, whether it is invasive, and more [68]. The higher the requirements, the tighter the controls applied to the device. If required, conformity assessment needs to be undertaken by a certified entity designated by the national regulatory body.

For all classes of device, the manufacturer is required to provide a technical file [68]. Therefore, having a quality management system (QMS) is one of the first steps to ensure you meet compliance [70]. The QMS for Medical Devices or ISO 9001 contains around fifty to several hundred procedures depending on the complexity of your product and process. Therefore, companies need the knowledge and experience to complete diverse reports and records, the Technical File for CE marking or 510 K for submission to the FDA for US markets [68,69].

None of the companies younger than five years considered it worth the effort. Most companies mentioned that understanding, meeting and measuring the regulation requirements translates into a cost AR companies cannot afford. For the companies, there is no transparent process to get their products certificated.

Therefore, instead of looking for medical certification, most companies only seek and achieve conformity with the CE marking standards that rule the use of products sold within the European Economic Area. To put this in perspective, this means most of these AR companies follow the same procedure as toy manufacturers. The practical implication of this choice is that doctors cannot prescribe toys and medical insurance do not recognise toys either. Therefore, the patient should have to cover the full cost of the product.

We mentioned in previous sections the lack of an appraisal tool. Existing legal and regulatory systems, initially designed for medicine, have not been adapted to the characteristics of AR. However, most importantly, they do not allow entrepreneurs and start-ups to enter and meet requirements without creating significant expenses.

### 4.4. Technical

This category discusses technological viability for AR in the healthcare market, not state of the art challenges. It includes barriers for the adoption due to the nature of these technologies.

#### Skilled Health Personnel and System Constraints

There appears to be a gap in the provision of education and training of healthcare professionals regarding AR, as all the companies interviewed currently trading said they had to spend time and money training healthcare professionals and carers on how to use their products. It seemed that this could be addressed by improving the curriculum of healthcare staff.

This acts as one constraint for the adoption of new technologies. Integration problems, such as obsolete data records, or other current software being used as per regulatory requirements in health settings also slows down the development process of RAS. The lack of infrastructure was also a concern; the availability of resources such as connectivity to the internet in remote healthcare organisations or directly with the final user, limit the market further.

### 4.5. Social, Cultural and Behavioural

This category describes the opposition from the healthcare professionals to AR, the willingness of the healthcare stakeholders to incorporate AR into their work environment and care of patients.

#### Consumers’ Acceptance and Ethical Concerns

Attitudes and beliefs were seen as a crucial barrier for the market, slowing the introduction of assistive robots in healthy environments. There is currently a distrust in AR companies, enhanced by a lack of understanding of how the final product works. This impacts on stakeholders’ understanding of the benefits and opportunities from AR.

The current distrust is also driven by concerns over patient safety, but also strong resistance from staff to change their current practices, considering AR will disorder the delivery of care. Some concerns were also raised about the belief among healthcare professionals that robots will take their jobs.

Furthermore, ethical concerns were mentioned concerning AR and its underpinning technology, to include robots’ levels of autonomy, and more general concerns around automation. This fear of automation and AR is leading to an industry that is technology lagging and consequently, the sector and crucially patients are missing out on these advances. The consequence for eHealth start-ups is that they have to commit their resources, to face the market misconceptions of AR.

Education is a critical aspect. Like many other implementation evaluation studies [71], better education has been seen as a facilitator for the eHealth market. Skills-related barriers, such as healthcare professionals’ and end-users’ technological abilities and experience, also influence the implementation and acceptance of AR.

## 5. Conclusions

Given the potential of assistive robotics (AR) to improve lives, and upon a backdrop of concerns around growing costs in health services, research on the adoption of AR in a health care setting is still minimal. There is a clear need for research into improving the efficiency of the AR marketplace [15]. The need to identify persisting innovation barriers in the health industry has also been recognised by the European Innovation Partnership. Thus, while the entry of AR into the mainstream healthcare sector has been constrained by a range of obstacles slowing down the adoption of the technology, to our knowledge, no research explores this issue specifically for this high-tech market.

The presented work presented new insight into and addressed the source and nature of the barriers that are inhibiting a more rapid and widespread adoption of advances in AR in healthcare.

Identifying market barriers has clear policy relevance. Addressing these barriers, policymakers could increase the entrepreneurship and boost the performance of the sector. Our goal is to raise awareness of these barriers, that further research may be conducted and that policymakers may develop a sustainable framework that ensures AR and associated technologies are realised in the healthcare sector.

Some of the main findings are in agreement with previous research that identifies barriers in the adoption of digital technologies in eHealth and the co-evolutionary and path-dependent nature of the innovation process more generally [5]. However, the impact of our identified barriers in the AR market, and crucially, their interaction, is more far-reaching than for digital companies in eHealth. Since this represents a new market for the healthcare sector, there are several policy gaps impacting the development process and the procurement and adoption of assistive robots.

There is a need to strengthen bonds to sources of specific and codified knowledge in more traditional industries [72]. Fostering a culture of collaboration, involvement, education, and communication have been seen as ways of overcoming healthcare stakeholders’ opposition to digital innovations [73,74]. The concerns about liability, patient privacy, and security, also mentioned in studies regarding digital technologies [75,76], could be addressed through the introduction of institutes and legislation, designed solely for AR development. This also involves making a transparent, affordable, and accessible certification process for assistive technologies.

All of these show that to address the significant barrier of access to the healthcare sector, governments have to take a more proactive role. To overcome market failure or inefficiencies that result from these barriers government intervention is vital [77]. From the creation of legislation and policies to the establishment of open standards for the development of AR, governments could sustainably facilitate the implementation of assistive robots in different healthcare environments. Governments can also subsidise AR and provide seed funding with positive externalities [78].

However, this governmental effort should be a joint effort among different countries. Supportive legislation to ease the adoption of innovation among healthcare systems from different countries should be encouraged. Only then can the real potential of AR be realised, providing the companies with new opportunities, expanding the size of their market. As a result, venture capital firms, angel investors, crowdfunding providers and other financial backers will start reducing their risk perception.

Although we presented a comprehensive evaluation of market barriers, further research is needed, in particular in other types of regions, including less developed economies.

## Figures and Tables

**Table 1:** Trading activities of assistive robotic companies contacted from February 2019 to March 2019. (Note: -- indicates that the company did not share the information, * year that the relevant robotics division was created).

Company	Year Founded	Total Funding Amount (* M$)	Product	Currently Trading
SONY	* 1990	--	AIBO	Worldwide
Intuition Robotics	2015	22	ElliQ	Pre-sale US
Blue Frog Robotics	2014	0.18	Buddy	No
Emotech LTD	2014	10	Olly	No
Jibo	2012	72.7	Jibo	No
Temi	2015	21	Temi	No
ASUS	--	--	Zenbo	No
Groove X	2015	52.7	Lovot	Japan
UBTech Robotics	2012	940	Lynx	US
Zoetic AI	2017	--	Kiki	No
Yukai Engineering	2011	--	BOCCO emo	Japan

**Table 2:** Assistive robotic companies interviewed. (Note: -- indicates that the company did not share the information).

No. of Employees	Registration	Age (Date Reported, Year)	Assets (Last Reported, 1000 Eur)	Currently Trading	Country	Product Description
1–10	July-18	0.24	5.6	No	UK	Wheelchair robotic arm
1–10	January-18	0.74	5.6	No	UK	Therapeutic robot
1–10	November-17	0.91	--	No	UK	Emergency drone
1–10	September-17	1.07	75	No	France	Medication reminder robot
1–10	August-17	1.16	--	No	UK	Care support robot
1–10	August-17	1.16	28.5	No	UK	Medication delivery robot
1–10	May-17	1.41	11.2	No	France	Smart wheelchair
11–20	September-16	2.07	155	Yes	France	Care support robot
1–10	September-16	2.07	5	No	France	Assistive robotic arm
1–10	July-16	2.24	192.5	Yes	France	Care support robot
1–10	April-16	2.49	143.7	Yes	France	Telepresence robot
--	January-16	2.74	--	No	France	Medication delivery robot
1–10	July-15	3.25	50	Yes	France	Patient monitoring solution
1–10	November-14	3.91	7.2	Yes	France	Therapeutic robot
30–50	November-12	5.91	50.3	Yes	France	Indoor projector robot
11–50	November-11	6.91	162.5	No	France	Care support robot
41–50	2007	11.00	15,400	Yes	France	Robotic air quality purification

**Table 3:** Barriers for AR companies.

Barrier Category	Barrier	Remarks
Market Failure	Access to the healthcare sector	*‘without contacts, there is not really a way into it’*
	Highly fragmented healthcare sector	*‘there are many different people involved’, ‘you can’t get to the people that make the choices’, ‘is quite hard to reach the client’*
	Poor market infrastructure	*‘you have to manufacture where the skills are’*
	Distrust in entrepreneurs	*‘people see us as buyers, instead of people trying to help others doing what we love’, ‘doors are not open to entrepreneurs with good ideas’*
	High investment requirements	*‘is not cheap, is an expensive journey’, ‘this stop people for doing, the cost puts an extra weight’*
Economic and Financial	High cost of capital	*‘bring a project together and fund that project is really really difficult’*
	Lack of/inadequate access to capital	*‘there are not investment opportunities for hardware’*
	High up-front capital costs for investors	*‘there is great risk involved in funding hardware companies’*
	Lack of access to credit for the consumer	‘[the product] *might be too expensive for the final user’, ‘you need to work on B2B’*
	Small market size	*‘we don’t know how the UK* [healthcare] *systems work’, ‘we will need someone to help us get to that market’*
Institutional	Lack of institutions and mechanisms	*‘there is a lack of directives’, ‘government support is minimal’*
	Lack of a legal/regulatory framework	*‘AI should be transparent’, ‘*[healthcare segment] *they are reluctant’*
	Lack of stakeholders’ involvement in decision making	*‘this is a problem, we got the solution, and no one listen* [to entrepreneurs]’
	Lack of universities’ participation	*‘you can’t put a price* [university support] *but, unfortunately, they are not interested in product development’*
	Lack of a clear certification process	*‘we cannot pursue a medical certification’*
Technical	Lack of skilled care personnel	*‘they haven’t seen a robot, so they don’t know how to use it’*
	Systems constraints	‘[challenge] *to know what technology to use’, ‘integrate all the technology is the main problem’*
Social, Cultural and Behavioural	Lack of consumer acceptance	*‘is quite hard to reach the client’, ‘is not here to take people jobs’*
	Unfounded moral and ethical concerns of AR	‘[invest time] *to convince people to have the robot’, ‘is not going to spy you’*

**Table 4:** Elements of identified barriers.

Barriers	Barrier Elements
***1. Market Failure***	
Access to the healthcare sector	Access to patients for product co-creation. Disrupts the whole development process.
Highly fragmented healthcare sector	Different stakeholders and organisations. Slows down technology acquisition.
Poor market infrastructure	Lack of manufacture opportunities in Europe. Increases final product cost and slows technology acquisition.
Distrust in entrepreneurs	Disrupts the development process and technology adoption.
High investment requirements	High seed funding needed to develop prototypes. Builds an entry barrier for entrepreneurs. Discourages entrepreneurs.
***2. Economic and Financial***	
High cost of capital	Fundamental differences between software and hardware investment requirements. Creates a lack of capital, high-interest rates, and risk perception by financial organisations. Impacts on economic viability.
Lack of/inadequate access to capital	No awareness of hardware development implications. Impacts market competition and market efficiency.
High up-front capital costs for investors	High seed funding need increases risk perception. Lack of understanding of AR investment needs.
Lack of access to credit for the consumer	High product cost. Under-developed credit market. Reduces market size.
Market size small	Fragment healthcare system between regions and countries. Prevents product scale and potential gains, reducing the appeal for entry of newcomers.
***3. Institutional***	
Lack of institutions and mechanisms	Missing agencies at the planning level to support AR development. Inhibits information dissemination between producers and consumers, creating extra costs for companies.
Lack of a legal/regulatory framework	Generates liability and concerns in the adoption of new technology.
Lack of stakeholders’ involvement in decision making	No seeking of the involvement of developers. Creates misplaced priorities, making policymaker bodies unaware of the market barriers.
Lack of universities’ participation	Impacts on recruitment and R&D opportunities.
Lack of a transparent certification process	No clear the path for certification of AR devices. Disrupts market entry of new products.
***4. Technical***	
Lack of skilled care personnel	Slows down technology adoption, creates extra expenses.
Systems constraints	Integration problems with healthcare IT infrastructure. Producers cannot realise the market.
***5. Social, Cultural and Behavioural***	
Lack of consumer acceptance	Fears surrounding the broader impact of AR, for example, fear of robots taking jobs. Reduces the market size.
Unfounded moral and ethical concerns of AR	Affects market size and technology adoption.

## Data Availability

Not applicable.
